# Vaginal microbiota in ethnically diverse young women who did or did not develop pelvic inflammatory disease: community-based prospective study

**DOI:** 10.1136/sextrans-2021-055260

**Published:** 2022-01-27

**Authors:** Sarah Kerry-Barnard, Liqing Zhou, Laura Phillips, Martina Furegato, Adam A Witney, S Tariq Sadiq, Pippa Oakeshott

**Affiliations:** 1 Population Health Research Institute, St George's, University of London, London, UK; 2 Applied Diagnostic Research and Evaluation Unit, St George's, University of London, London, UK; 3 Institute for Infection and Immunity, St George's, University of London, London, UK; 4 Clinical Academic group in Infection and Immunity, St George’s University Hospitals NHS Foundation Trust, London, UK

**Keywords:** pelvic inflammatory disease, microbiology, cohort studies, epidemiology, RNA

## Abstract

**Objectives:**

A lactobacilli-dominated vaginal microbiome may protect against pelvic inflammatory disease (PID), but one dominated by *Gardnerella* species might increase susceptibility. Not all lactobacilli are equally protective. Recent research suggests that D(−) isomer lactic acid producing lactobacilli (*Lactobacillus crispatus, Lactobacillus jensenii* and *Lactobacillus gasseri*) may protect against infection with *Chlamydia trachomatis*, an important cause of PID. *Lactobacillus iners* , which produces L(+) isomer lactic acid, may be less protective. We investigated the microbiome in stored vaginal samples from participants who did or did not develop PID during the prevention of pelvic infection (POPI) chlamydia screening trial.

**Methods:**

Long-read 16S rRNA gene nanopore sequencing was used on baseline vaginal samples (one per participant) from all 37 women who subsequently developed clinically diagnosed PID during 12-month follow-up, and 111 frequency matched controls who did not, matched on four possible risk factors for PID: age <20 versus ≥20, black ethnicity versus other ethnicity, chlamydia positive versus negative at baseline and ≥2 sexual partners in the previous year versus 0–1 partners.

**Results:**

Samples from 106 women (median age 19 years, 40% black ethnicity, 22% chlamydia positive, 54% reporting multiple partners) were suitable for analysis. Three main taxonomic clusters were identified dominated by *L. iners, L. crispatus* and *Gardnerella vaginalis*. There was no association between a more diverse, *G. vaginalis* dominated microbiome and subsequent PID, although increased Shannon diversity was associated with black ethnicity (p=0.002) and bacterial vaginosis (diagnosed by Gram stain p<0.0001). Women who developed PID had similar relative abundance of protective D(−) isomer lactic acid producing lactobacilli to women without PID, but numbers of PID cases were small.

**Conclusions:**

In the first-ever community-based prospective study of PID, there was no clear association between the vaginal microbiome and subsequent development of PID. Future studies using serial samples may identify vaginal microbial communities that may predispose to PID.

## Background

Pelvic inflammatory disease (PID) is an infection-induced inflammation of the female upper reproductive tract that can lead to infertility and ectopic pregnancy. *Chlamydia trachomatis* and *Neisseria gonorrhoeae* cause up to 30% of PID, but in many cases, the aetiology is polymicrobial or unclear.^1^ In addition, we do not know why only some women with genital *C. trachomatis* infection develop upper tract infection. A healthy, low diversity, vaginal microbiome dominated by lactobacilli may protect against disease.^2–5^ However, a high diversity microaerophilic/anaerobic microbiome with fewer lactobacilli (as may be found in bacterial vaginosis often with *Gardnerella vaginosis* and/or *Atopobium vaginae*) may increase susceptibility to STIs by causing cervicomucosal barrier disruption and epithelial portals of entry for ascending infection.^3 6^

Not all lactobacilli are equal.^3^ A recent study suggested that lactobacilli that produce mainly D(−) isomer lactic acid (*Lactobacillus crispatus, L. jensenii and L. gasseri*) may be associated with long-term protection against *C. trachomatis* infection, possibly by reducing epithelial cell recycling.^7^ By contrast, a vaginal microbiome dominated by *L. iners,* which mainly produces L(+) isomer lactic acid, may be associated with increased susceptibility to infection.^7–9^

A recent systematic review called for longitudinal studies of vaginal microbiota and infections to lay the foundation for possible prevention and treatment strategies.^5^ We used stored baseline self-taken vaginal swabs from a cohort of 2357 ethnically diverse young female students recruited from public areas at 22 London colleges to the prevention of pelvic infection (POPI) chlamydia screening trial in 2004–2006.^10^Previously, 16S rRNA sequencing of 20 samples using a long-read PacBio sequencing platform had confirmed adequate DNA integrity in these samples.^11^

Conventional short-read 16S rRNA gene sequencing is constrained in its ability to comprehensively speciate pathogens within a sample because of the limited 16S variable regions that are usually sequenced. Long-read sequencing platforms such as PacBio and Oxford Nanopore Technologies’ MinION allow for full sequencing of the 16S rRNA gene for microbiome studies.^12 13^

We used long-read 16S rRNA gene nanopore sequencing to investigate bacterial communities in 37 women who developed clinical PID within the following year, and 111 frequency matched control women who did not. Since D(−) isomer lactic acid producing lactobacilli may protect against *C. trachomatis*,^5 7^ a major cause of PID, we also explored the relative abundance of D(−) isomer lactic acid producing lactobacilli in women with and without subsequent PID, and in women with and without concurrent *C. trachomatis* infection or bacterial vaginosis (BV).^14^

## Methods

### Study samples

**Figure 1:**
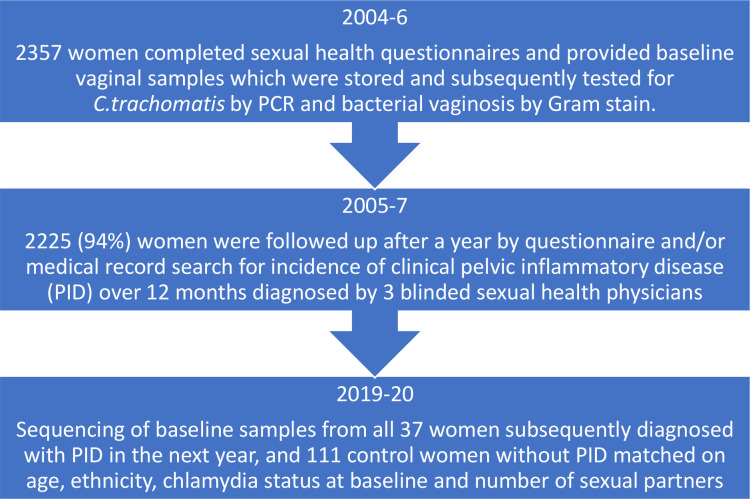
POPI vaginal microbiota study flow chart. PID, pelvic inflammatory disease; POPI, prevention of pelvic infection.

One hundred and forty-eight frozen, baseline vaginal samples (one sample per participant) were retrieved from all 37 women who subsequently developed clinically diagnosed PID in the next year and 111 controls with no PID, as part of the POPI trial^10^ (figure 1). At recruitment, participants had provided self-taken vaginal swabs in the college toilets, rolled the swab over a glass slide for future Gram stain analysis for BV (Nugents score ≥7) and placed the swab in a tube of Aptima transport medium (Gen Probe), which was frozen within 24 hours. Controls (three per index case of PID) were frequency matched on possible risk factors for PID^15^: age <20 versus ≥20, black ethnicity versus other ethnicity, chlamydia positive versus negative at baseline and ≥2 sexual partners in the previous year versus 0–1 partners. The diagnosis of PID was made by three sexual health physicians (blinded to chlamydia status) based on medical records and participant questionnaires after 12 months and using modified Hager’s criteria: pelvic pain, cervical motion tenderness and/or adnexal tenderness.^10^

### DNA preparation and nanopore sequencing

DNA was isolated from vaginal samples using QIAamp DNA Mini Kit (Qiagen), following the manufacturer’s protocol for Gram-positive and difficult-to-lyse bacteria, and quantified using Qubit 3.0 Fluorometer. 16S rRNA genes in each sample were amplified with the primers, B27F (TTTCTGTTGGTGCTGATATTGCAGRGTTYGATYMTGGCTCAG) and B1492R (ACTTGCCTGTCGCTCTATCTTCRGYTACCTTGTTACGACTT), which were modified from S-D-bact-0008-c-S20 and S-*-Univ-1492-a-A-19.^16^ DNA libraries, each containing 12 different clinical samples, were prepared using PCR Barcoding Kit - EXP-PBC096 and Ligation Sequencing Kit 1D - SQK-LSK108, and sequenced on MinION MK I with SpotON Flow Cell Mk I FLO-MIN106 R9.4 (Oxford Nanopore Technologies). Sequencing reads were live base called locally using the MinKNOW protocol - NC_48Hr_Sequencing_Run_FLO-MIN106_ SQK-LSK108.py. Sequence data have been submitted to the ENA database with accession number PRJEB41336.

### Microbiome comparison and statistical analysis

The sequence analysis protocol was based on previously published methods.^17^ Briefly, reads were first filtered for length >1200 and <1800 bases, before chimeric reads were removed with yacrd.^18^ Remaining reads were mapped to the National Center for Biotechnology Information (NCBI) 16S Microbial database using minimap2.^19^ Aligned reads were merged by species using R, counted and normalised using decostand (R vegan package V.4.0.4, 2021-02-15) by dividing by the margin total. The normalised number of reads mapping to each species against that sample was shown in a heat map. Shannon alpha diversity metrics were generated with vegan, and statistical significance was determined using a Kruskal-Wallis rank sum test for six clinical characteristics: PID versus no PID, age <20 versus ≥20, black ethnicity versus other ethnicity, chlamydia positive versus negative at baseline, ≥2 sexual partners in the previous year versus 0–1 partners and bacterial vaginosis by Gram stain versus no bacterial vaginosis. P values were adjusted for multiple comparisons using the Benjamini-Hochberg method.

The relative abundance of the dominant species was explored (including a combination of D(−) isomer lactic acid producing lactobacilli *L. crispatus, L. jensenii* and *L. gasseri*), for three clinical diagnoses: pelvic inflammatory disease, bacterial vaginosis or *C. trachomatis*. Finally, we investigated beta diversity using non-metric multidimensional scaling (NMDS) of Bray-Curtis distances.

The study was constrained by the number of women diagnosed with PID. We estimated that a sample size of 148 women (37 who developed PID and 111 who did not develop PID) would detect a significant difference (p<0.05, power 80%) if vaginal samples from 35% of women with PID had *Gardnerella vaginalis* 15 versus ≤11% of women without PID. (We used *G. vaginalis* as a surrogate for bacterial vaginosis.)

### Excluded samples

An initial heatmap representation of the relative abundances (online supplemental figure 1) showed four major clusters, one of which was dominated by the plant pathogen *Burkholderia gladioli*. Although a rare cause of opportunistic infection in humans,^20^ the presence of *B. gladioli* is unusual, and the *Burkholderia* genus has been associated with contamination in other microbiome studies.^21^ Further investigation showed that the majority of samples with high levels of *B. gladioli* were collected within a specific time period at the end of the study (online supplemental figure 2), suggesting a systematic contamination within the samples collected at the time. A similar plot for *L. crispatus* showed no chronological bias (data not shown). In light of this, all 42 samples containing more than 1% *B. gladioli* were removed from further analysis. (This included 14 samples with PID, 13 with BV and 9 with *C. trachomatis*.) Inclusion or exclusion of these samples had little effect on the conclusions of this study.

10.1136/sextrans-2021-055260.supp1 Supplementary data

## Results

### Study population

Samples from 106 participants (23 with PID) had adequate sequencing data and were included in the analysis. The median age of the women in the cohort was 19 years (range 16–27), 40% (42) described their ethnic group as black and 54% (57) reported ≥two sexual partners in the previous year. At baseline, 22% of women (23) had *C. trachomatis*, 21% (22) had bacterial vaginosis (diagnosed by Nugent’s score 7–10 on Gram stain, with a further three classified as intermediate), 4% (4) had *Mycoplasma genitalium* and 2% (2) *Neisseria gonorrhoeae* (diagnosed by Gen-Probe PCR). The estimated mean time from baseline sample to diagnosis of PID, where data were available (in 22 of 23 women), was 35 weeks (range 13–52 weeks).

### Vaginal microbiota show three main clusters

**Figure 2:**
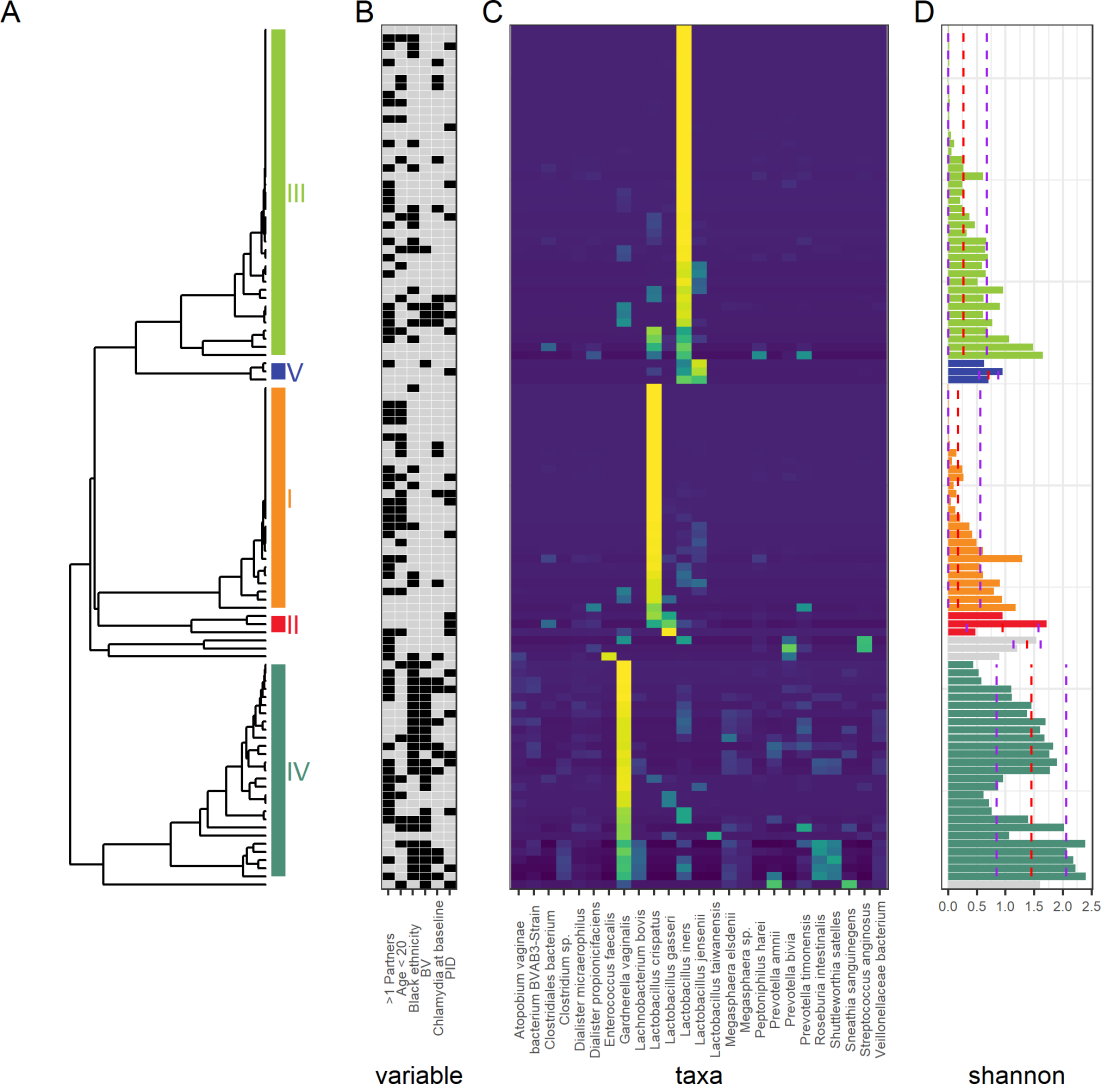
Heat map of relative abundances of microbial taxa in the vaginal bacterial communities of 106 women who did or did not develop pelvic inflammatory disease (PID) in the next 12 months. (A) Hierarchical clustering based on the Pearson correlation dissimilarity of the relative abundances within each sample. Clusters are labelled I-V^16^. (B) Clinical characteristics: black filled box indicates PID, Chlamydia positive at baseline, >1 sexual partner in the past year, age < 20 years, self-assigned black ethnic group. (C) Heatmap of relative abundances showing only the most common species. (D) Shannon diversity for each sample: red dashed line shows the median diversity for each cluster, purple dashed lines show ± the SD.

Figure 2 shows three main taxonomic clusters dominated by *L. iners, L. crispatus* and *G. vaginalis,* with a smaller number of samples dominated by *L. jensenii* and *L. gasseri*. The lactobacillus dominated clusters showed lower Shannon diversity than the *G. vaginalis* cluster.

### No association between increasing diversity and PID

**Figure 3:**
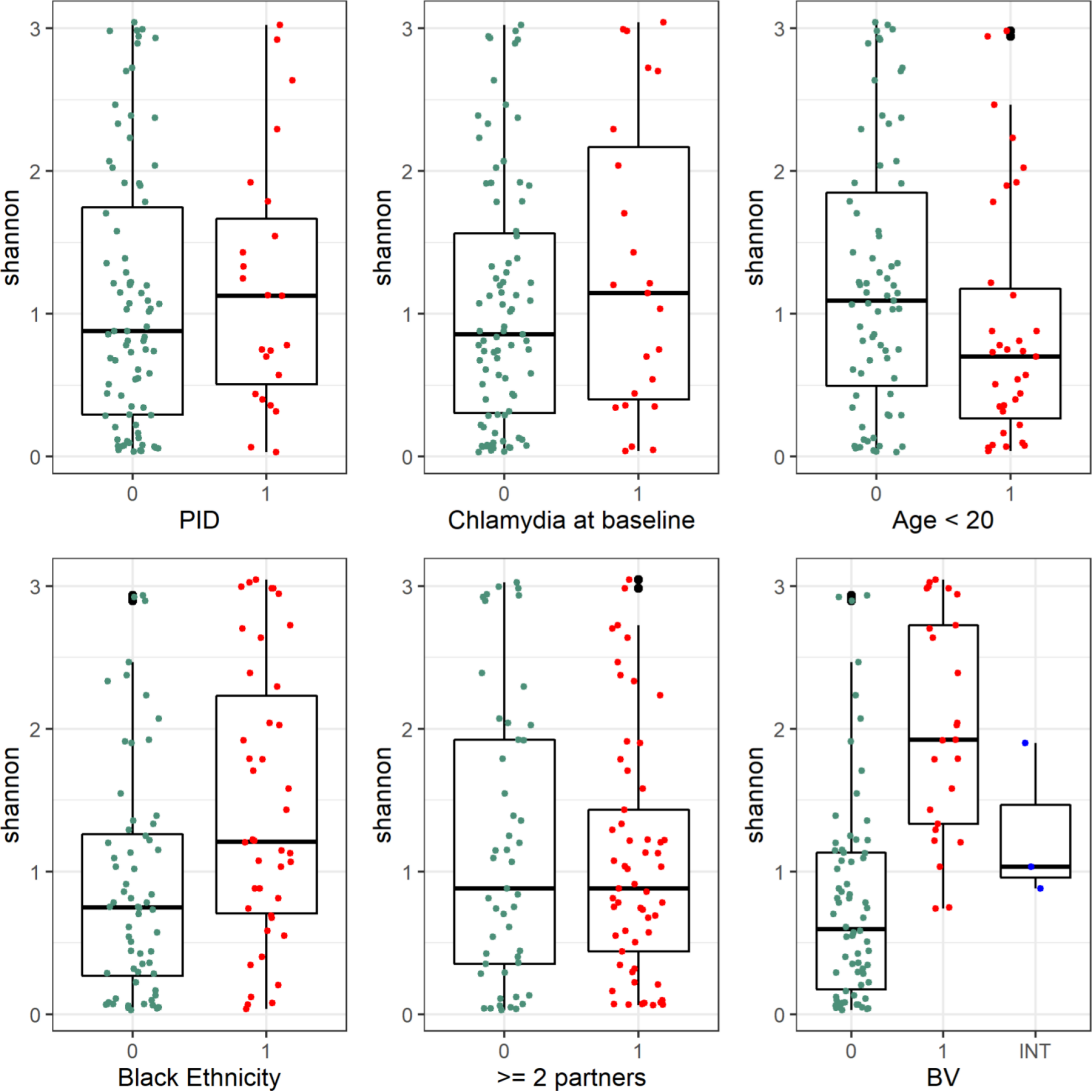
Shannon diversity plotted for each clinical risk factor. Kruskal-Wallis rank sum test shows differences in Shannon diversity between groups for bacterial vaginosis (BV; n=22, p=5.4 ×x10^-8^), black ethnic group (n=42, p=0.002), age <20 (n=33, p=0.048 less diverse), but no differences for pelvic inflammatory disease (n=23, PID: p=0.53), Chlamydia at baseline (n=23, p=0.38), ≥2 sexual partners (n=57, p=0.88). 1 (red)=Has risk factor, 0 (green)=does not have risk factor.

Comparing Shannon alpha diversity (figure 3) across six risk factors (PID, *C. trachomatis,* BV, age, ethnicity and multiple partners) showed no association between a more diverse microbiome and subsequent PID (Kruskal-Wallace rank sum test p=0.53) or concurrent *C. trachomatis* infection (p=0.38). However, there was a significantly higher diversity in samples from participants aged ≥20 years (p=0.048), participants of black ethnic group (p=0.002) and those with a diagnosis of bacterial vaginosis (BV, p<0.0001).

### PID was not associated with lower abundance of D(−) isomer lactic acid producing lactobacilli

**Figure 4:**
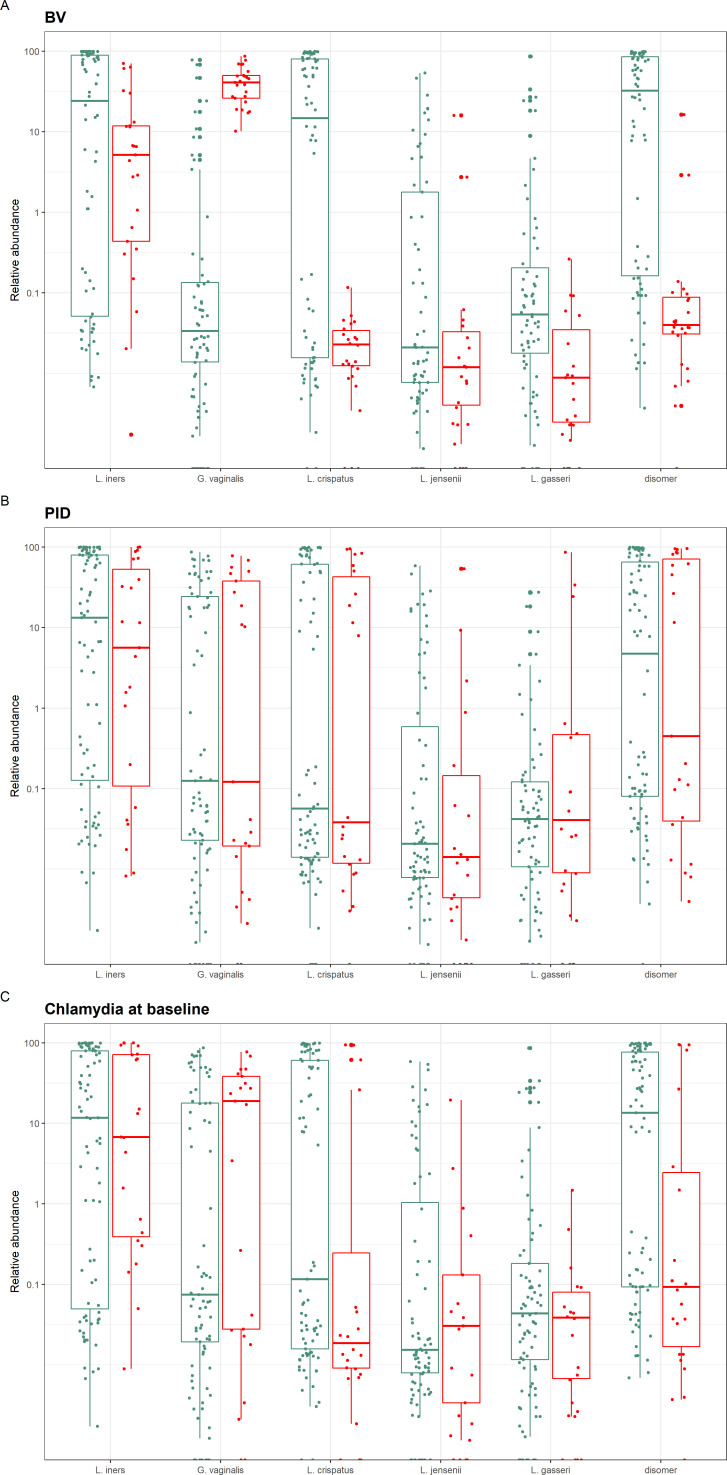
Relative abundance of the dominant species, including a combination of D(−) isomer lactic acid producing lactobacilli (*Lactobacillus crispatus, L. jensenii* and *L. gasseri*), compared for the three main clinical diagnoses: box A: bacterial vaginosis (BV); box B: pelvic inflammatory disease (PID); box C: chlamydia trachomatis at baseline. Red: has clinical diagnosis. Green: does not have clinical diagnosis. BV (A) was associated with lower abundances of D(−) isomer lactic acid producing lactobacilli species (p=4.05×10^-6^) and higher abundance of *Gardnerella vaginosis* (p = 3.06×10^-9^), but this did not apply to PID (B) or Chlamydia.

Figure 4 demonstrates the relative abundance of the five main species *Lactobacillus iners, Lactobacillus crispatus, Gardnerella vaginalis, Lactobacillus jensenii* and *Lactobacillus gasseri* (including a combination of D(−) isomer lactic acid producing lactobacilli *L. crispatus, L. jensenii* and *L. gasseri*), related to the three main clinical diagnoses:

1. BV at baseline.
2. PID within the next 12 months.
3. *C. trachomatis* at baseline.

This shows that unlike BV, neither PID nor *C*. t*rachomatis*, was associated with lower abundance of D(−) isomer lactic acid producing lactobacilli species (p=0.77 and 0.33, respectively). Online supplemental figure 3 shows similar results.

### Beta diversity shows no association with PID

Visualisation of Bray-Curtis based NMDS dimensions one and two (stress=0.09 after 20 iterations (online supplemental figure 4) showed no association with subsequent PID or concurrent *C. trachomatis*. However for BV, three clusters correlated with the dominant species shown in figure 1 (*L. iners, L. crispatus* and *G. vaginalis),* with dimensions one and two distinguishing BV versus no BV (online supplemental figure 4A, B, respectively).

## Discussion

### Principal findings

In the first ever community-based, frequency-matched prospective study of vaginal microbiota in women who did or did not develop PID, we found no association between a more diverse microbiota and subsequent pelvic inflammatory disease. Women with bacterial vaginosis had lower relative abundance of protective D(−) isomer lactic acid producing lactobacilli, but this did not apply to women who developed PID.

### Strengths and weaknesses

This study is unique as it is the only community-based prospective study of the vaginal microbiome and PID, and the first to explore the possible association of PID with low levels of vaginal D(−) isomer lactic acid producing lactobacilli. Previous studies were based in hospitals or clinics,^5 9 22^ or were cross-sectional rather than prospective.^23^ Forty per cent of participants were of black ethnicity, a group who may have higher rates of bacterial vaginosis^8 24^ and STIs^10^ compared with other ethnic groups. This is supported by our finding that the *G. vaginalis* taxonomic cluster was associated with black ethnicity, an association that lends internal validity to this study.^8^ The project also responds to recent calls for vaginal microbiome research to include prospective studies^5^ and data on sexual behaviour.^2^

To our knowledge, this is also the first study to use long-read sequencing on self-taken vaginal swabs. Combined with evidence that unsupervised self-collected vaginal swabs give similar microbiota results to clinician-collected swabs,^25^ this study supports feasibility of larger scale community-based projects. Encouragingly, the clusters we identified using long-read sequencing were similar to those found in earlier studies^9 24^ with three main clusters: one dominated by *L. iners*, one by *L. crispatus* and a more diverse cluster dominated by anaerobes. Further strengths include our pilot work confirming the integrity of stored samples^11^ and the availability of detailed information on the demographics and baseline infection status of all participants, with sequence analysis being conducted blind to these characteristics. Finally, the large number of participants (>2300) in the POPI trial ensured that controls were well matched.

The main limitation is that this was an exploratory study with a small number of PID cases. However, it is similar in size to earlier studies^7 9 23^ and contributes to the very limited available data on vaginal microbiota composition in relation to PID. The small sample size also meant we could not do complex epidemiological analyses. Another major weakness is the absence of negative and positive controls. The lack of laboratory negative extraction controls meant we could not be sure that *B. gladioli* was a contaminant. Removal of affected samples did not seem to influence the results, although it did reduce the power of the study. PID is a clinical diagnosis of low specificity, with around a third of diagnoses not confirmed on laparoscopy. This could also weaken the power of the study. However, diagnosis was confirmed by three experienced sexual health physicians.^10^ Although we found a significant association between BV and lower abundance of D-isomer lactic acid producing lactobacilli, we could not attribute causality or measure D(−) isomer lactic acid concentrations in these samples. We only assessed samples at one time point, and there was an estimated time gap of 3–12 months between obtaining the samples and diagnosis of PID, with only two women diagnosed with PID within 4 months of sampling. The vaginal microbiota may have changed over time in a significant proportion of women, potentially weakening the power of the study.^6^ However, this was unavoidable in a prospective study of PID.^22^

Another weakness is that the sensitivity of samples stored for 16 years may be reduced. Nonetheless, other sequencing studies using stored samples have shown reliable results.^11 26^ Although commonly used, the Kruskal-Wallis rank sum test is not generally considered appropriate for differential analysis of compositional data. A differential abundance analysis approach that is suited to compositional data would have been preferable. We used 16S rRNA gene sequencing, which does not cover fungi, protozoa and viruses, and may not reliably identify low loads of *C. trachomatis* or *N. gonorrhoeae*.^3^ However, samples had already been tested for *C. trachomatis, N. gonorrhoeae* and *M. genitalium* by PCR.^10 15^ We could not evaluate three recently identified novel *Gardnerella* species,^27^ as it is unclear whether full length 16S rRNA sequencing adequately differentiates the species within the *Gardnerella* genus, and they are not yet included in NCBI and other 16S rRNA databases. In addition, the taxon for bacterial vaginosis associated bacteria such as BVAB-1 (*Candidatus Lachnocurva vaginae*) may be misclassified as *Shuttleworthia* using the NCBI database. Finally, we analysed by the dominant species within a community state type^9^ rather than by actual community state type,^28^ but this would likely give similar results.

### Comparison with other studies

There is a dearth of studies of vaginal microbiota in women with and without PID,^3 23 29^ and like ours, none have shown a clear relationship between the vaginal microbiome and subsequent PID. A cross-sectional study from China used high-throughput sequencing on pelvic and cervical samples from 38 women with PID and 19 controls. They found microbiota in PID could be dominated either by a single organism or by polymicrobial infection.^23^ Analyses of bacterial vaginosis and incident PID in the Gynecologic Infection Follow-Through study were also inconclusive, with one analysis showing no association^22^ and others that women with bacterial vaginosis-associated bacteria had increased risk of PID.^6 14^ Interestingly, microbiological analysis of POPI samples^15^ found bacterial vaginosis was not significantly associated with PID after adjustment for baseline *C. trachomatis* infection. Most recently, Trent and colleagues analysed samples from 26 women aged 13–25 years with PID who were enrolled in the Technology Enhanced Community Health Nursing study.^29^ Over half of participants had low abundance of Lactobacillus species indicative of bacterial vaginosis.

Other studies of the vaginal microbiome in healthy ethnically diverse women have found similar main clusters/community state types to those in our study, two dominated by lactobacilli and one with higher proportions of anaerobic organisms.^9 24^ A recent study of the vaginal microbiome during genital infections^4^ found that *L. crispatus* was progressively replaced by *L. iners* in the shift from a healthy to an infected microbiome. As in our study, this was mainly characterised by anaerobic genera such as *Gardnerella, Prevotella, Megasphaera, Roseburia* and *Atopobium*. We did not find an association between *C. trachomatis* and *L. iners* dominated vaginal microbiota,^9^ but numbers with *C. trachomatis* infection were small. Finally, our finding of lower rates of protective D(−) isomer lactic acid producing lactobacilli in women with (vs without) concurrent bacterial vaginosis confirms other studies.^7^ The cervicovaginal microbiota could modulate host functions to protect against infection.^7^

### Implications

This study supports the feasibility of larger scale community-based projects, the use of self-collected vaginal swabs and the integrity of stored samples. The significantly higher abundance of D(−) isomer lactic acid producing lactobacilli in women without bacterial vaginosis is in line with other studies suggesting a protective role for these lactobacilli.^3 7 30^ Finally, negative studies are important. The key impact from this study is the need for more work, particularly serial sampling studies, to gain knowledge of predisposing vaginal communities that may lead to PID.

**Key messages**

- There are few data on vaginal microbiota and subsequent development of pelvic inflammatory disease (PID).
- A lactobacilli-dominated vaginal microbiome may protect against pelvic inflammatory disease, but one dominated by *Gardnerella* species might increase susceptibility.
- In this first ever community-based prospective study of PID, there was no clear association between vaginal microbiota and development of PID in the next 12 months.
- In line with previous studies, findings highlight the need for large serial sampling studies to identify vaginal microbiota that might predispose to PID.

## Data Availability

All data relevant to the study are included in the article or uploaded as supplemental information. Not applicable.
