# Supplementary material for: Vaginal microbiota in ethnically diverse young women who did or did not develop pelvic inflammatory disease: community-based prospective study
Source: Sex Transm Infect. 2022 Jan 27;98(7):503–9. doi: 10.1136/sextrans-2021-055260 (PMC9613871; doi:10.1136/sextrans-2021-055260)

Supplementary Figure 1. Heat map of relative abundances of microbial taxa in the vaginal bacterial communities of 148 women who did or did not develop pelvic inflammatory disease (PID) in the next 12 months.

A) Hierarchical clustering based on the Pearson correlation dissimilarity of the relative abundances within each sample. Clusters are labelled I-V according to those defined in Ravel et al [16] with the addition of a ***B. gladioli*** cluster (BG).

B) Clinical characteristics: Black filled box indicates PID, Chlamydia positive at baseline, >1 sexual partner in the past year, Age < 20 years, Self-assigned black ethnic group

C) Heatmap of relative abundances showing only the most common species

D) Shannon diversity for each sample: red dashed line shows the median diversity for each cluster, purple dashed lines show +- the standard deviation

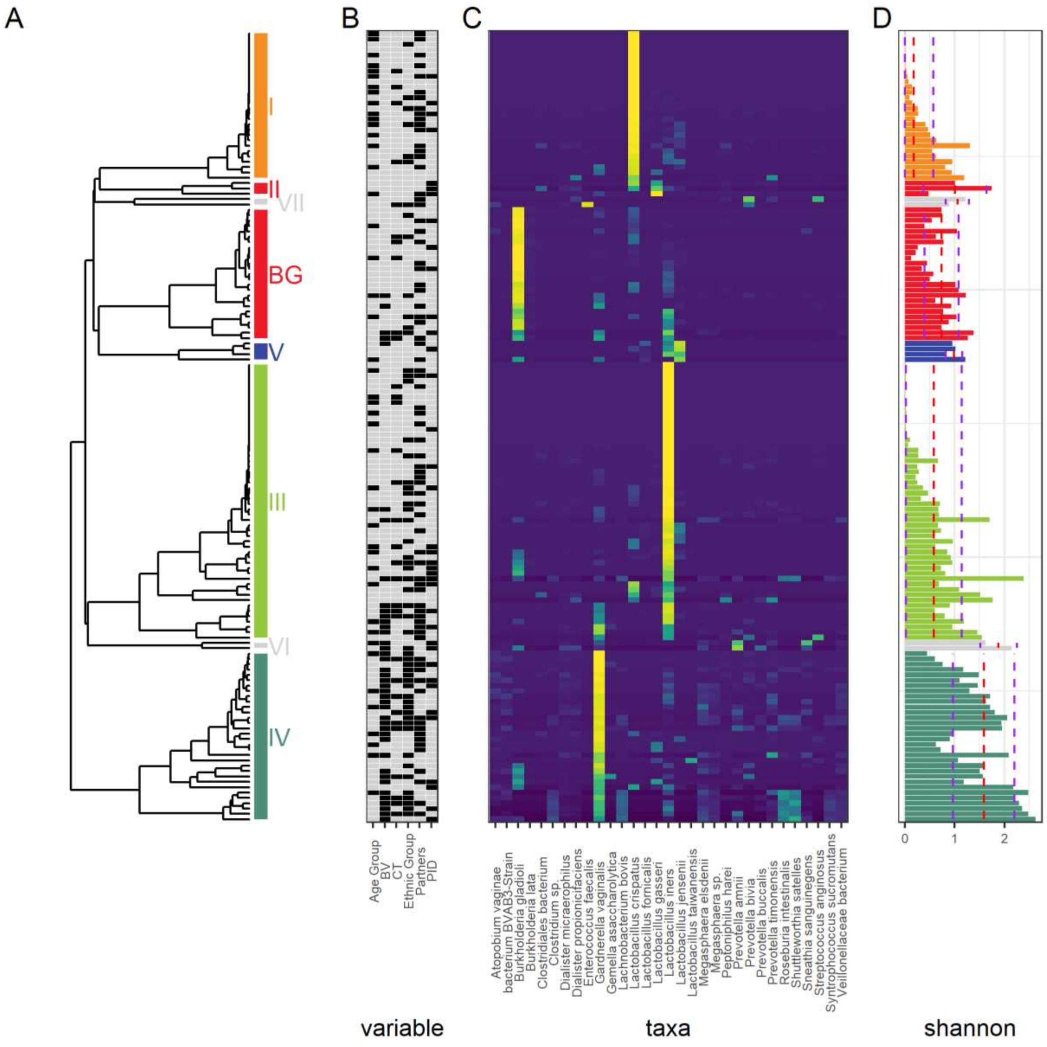

Supplementary Figure 2. Relative abundance of *B. gladioli* for each sample, ordered by the date of swab collection. Blue lines separate years of the study. The red line shows that swabs collected after March 2006 had a high percentage of *B. gladioli* representative sequences in the samples suggesting this was a contaminant.

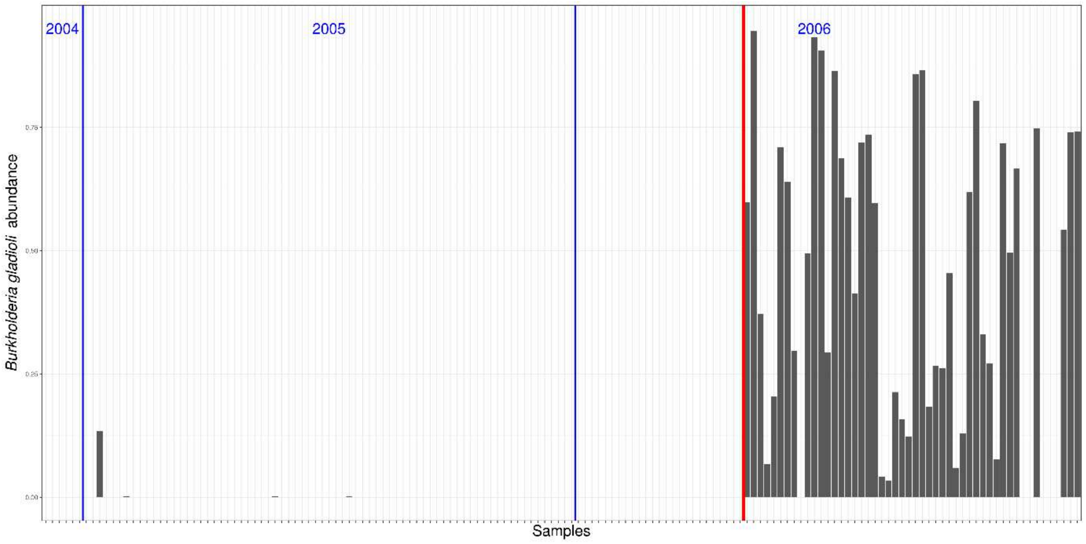

Supplementary Figure 3. Relative abundance of the dominant species in the three major clusters compared for each risk factor.

A) PID, B) Chlamydia at baseline, C) Age < 20, D) Black ethnicity, E) ≥2 sexual partners , F) BV at baseline

Red: Has risk factor. Green: Does not have risk factor

BV is associated with low levels of *L. crispatus* (p = 3.96x10^{-3}) and high levels of *G. vaginalis* (p = 3.06x10^{-9} Box F). *G. vaginalis* is also associated with black ethnicity (p = 0.004 Box D)

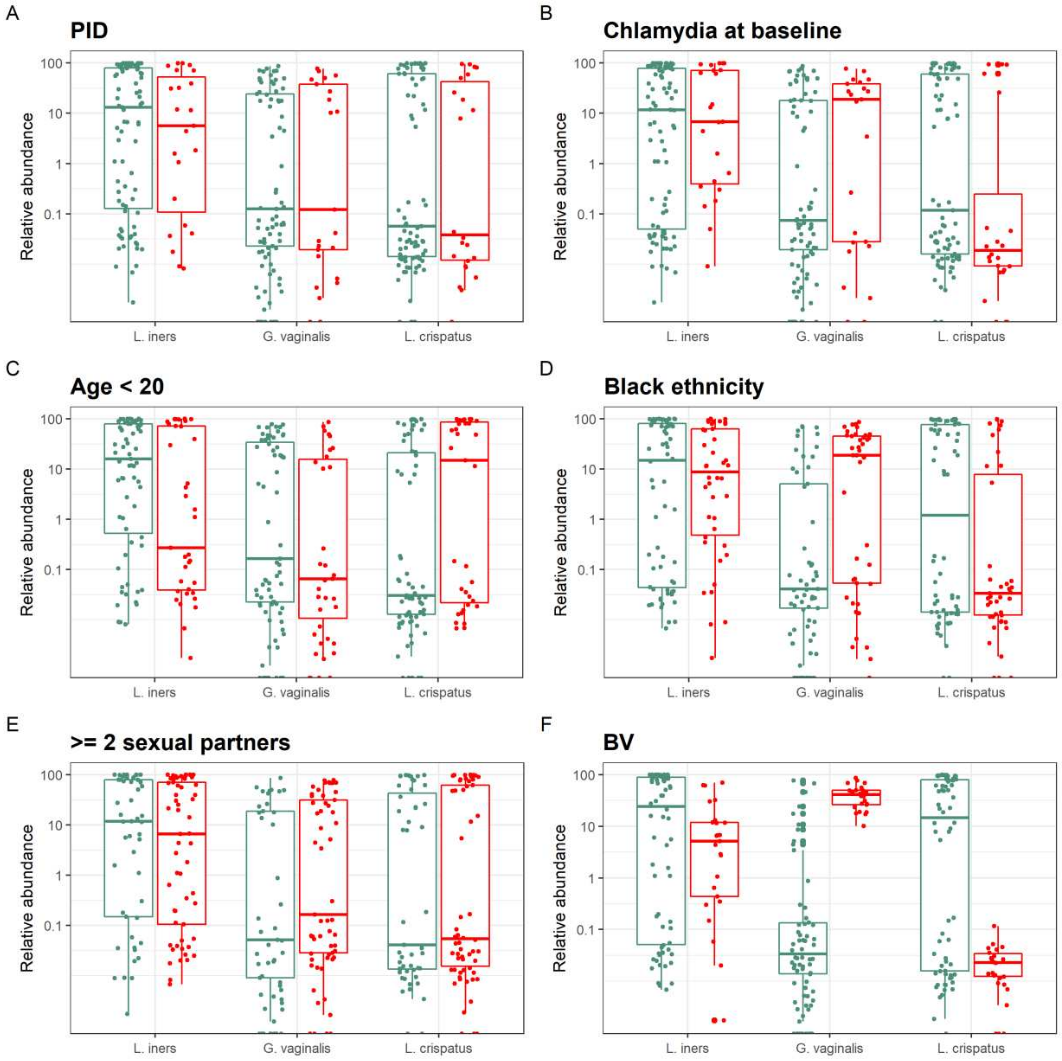

Supplementary Figure 4. Non Metric Multi-dimensional Scaling (NMDS) of Bray-Curtis distances for Bacterial vaginosis (BV), Chlamydia at baseline, and Pelvic inflammatory disease

Coding: Boxes A and B **BV** (red), no BV (green) or intermediate BV (blue). Box C **PID (pelvic inflammatory disease):** Red subsequent PID, Green no PID. Box D **Chlamydia**: Red Chlamydia positive, Green no chlamydia.

Box A Species fitted; Boxes B, C and D Ellipses drawn at the SD of the mean of each group.

For Bacterial vaginosis (Boxes A and B) the three clusters correlated with the dominant species in Figure 1: *L. iners, L. crispatus, G. vaginalis.* Similarly dimensions 1 and 2 distinguish BV versus no BV (Box B), but this did not apply to PID (Box C) or to Chlamydia (Box D).

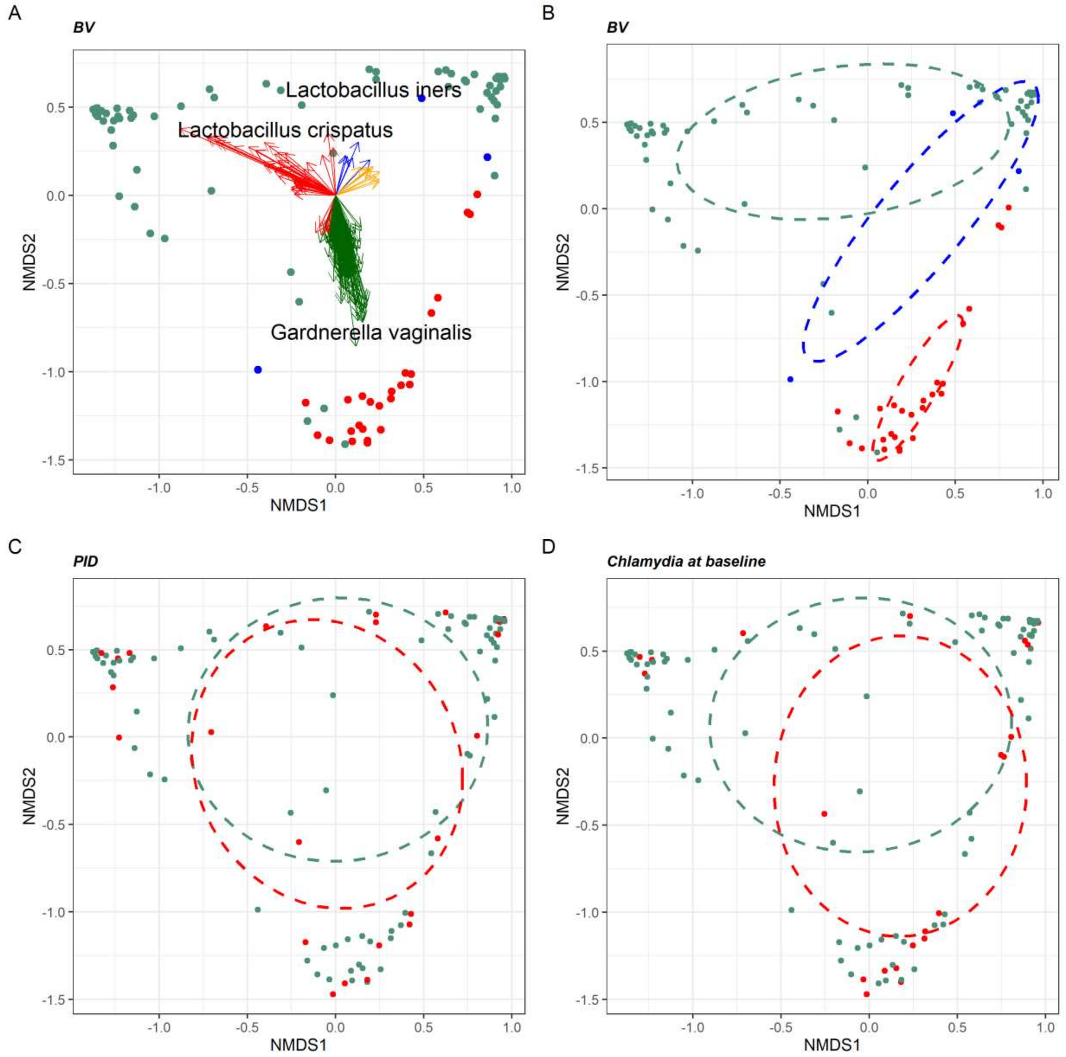

Supplement: Supplementary data [file sextrans-2021-055260supp001.pdf]
